# Comparative LC‐MS Proteomics of Quinoa Grains: Evaluation of Bioactivity and Health Benefits by Combining In Silico Techniques With In Vitro Assays on Colorectal Adenocarcinoma Cells

**DOI:** 10.1002/mnfr.70125

**Published:** 2025-05-23

**Authors:** Alessandro Zaccarelli, Beatrice Mattina, Rocío Galindo‐Luján, Laura Pont, Fernando Benavente, Ilaria Zanotti, Lisa Elviri

**Affiliations:** ^1^ Department of Food and Drug University of Parma Parma Italy; ^2^ Department of Chemical Engineering and Analytical Chemistry Institute for Research on Nutrition and Food Safety (INSA·UB), University of Barcelona Barcelona Spain; ^3^ Serra Húnter Program, Generalitat de Catalunya Barcelona Spain

**Keywords:** bioactivity, data analysis, mass spectrometry, peptides, proteomics, quinoa

## Abstract

In the present study, we investigated the potential biological effects of proteins and peptides extracted from four different commercial quinoa grain varieties: Black (B), Red (R), White (W), and Royal White (RO), using Caco‐2 cells as a proliferation model. Concentration–response curves were constructed to evaluate cytotoxicity and antiproliferative effects. Interestingly, peptides exhibited higher cytotoxicity than proteins, particularly in R and RO quinoa varieties. Based on these findings, we used a data mining approach to identify and compare the cytotoxic proteins in the four quinoa varieties. Using their relative abundance, we successfully classified R and RO quinoa as a cohesive group using classification models. To gain deeper insights into the biological effects on cells, we comprehensively analyzed the differential expression of apoptosis biomarkers using liquid chromatography‐tandem mass spectrometry (LC‐MS/MS) targeted proteomics. Finally, we correlated the apoptosis profile with the relative abundance of cytotoxic proteins. All these studies were supported by the application of multivariate data analysis. The results demonstrated the potential anticancer of quinoa grain proteins and peptides and provided the basis for more focused mechanistic studies aimed at developing functional foods and nutraceuticals.

## Graphical abstract

Identifying health‐promoting molecules such as bioactive peptides in complex food mixtures is a major challenge. In this study, we investigated proteins and protein hydrolysates from four quinoa varieties to evaluate their potential anticancer effects on colorectal adenocarcinoma cells. Using a multidisciplinary approach combining in vitro assays, LC‐MS proteomics, and data mining, we identified peptide sequences with possible proapoptotic activity. Data analysis revealed a correlation between these peptides and the expression of apoptosis biomarkers, helping to better understand their biological role and facilitating the search for bioactive compounds in complex natural sources.

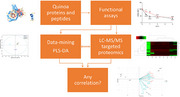

## 1 Introduction

Quinoa (*Chenopodium quinoa* Willd.) is an Andean grain that has garnered significant attention in recent years for its potential health benefits and nutritional value, including a high content of proteins with a well‐balanced amino acid profile [1, 2]. Despite being well‐known that quinoa consumption contributes to well‐being, special efforts are currently being made to obtain information about the potential effects of quinoa ingredients [2], such as proteins presenting antihypertensive [3], antidiabetic [4], and anticancer [5] bioactivities. Seeking to uncover its biological effects, our study delves into proteomics, functional assays, and data mining to comprehensively explore the potential antiproliferative properties of proteins and peptides derived from four distinct varieties of quinoa grains commercialized as Black (B), Red (R), White (W) (from Peru), and Royal white (RO) (from Bolivia). Previous research has laid the foundation for our investigation by establishing comprehensive protein maps of these quinoa varieties by label‐free liquid chromatography‐tandem mass spectrometry (LC‐MS/MS) shotgun proteomics [6]. Additionally, a data mining strategy has been developed to explore the immunonutritional bioactivity of quinoa proteins in silico [7], while other in vitro studies have identified quinoa as a potential source of bioactive peptides with anticancer activity [5]. Building on this, we investigated the effects of quinoa‐derived proteins and peptides on colorectal adenocarcinoma cells (Caco‐2), a widely used model for epithelial proliferation in cancer research [8]. Colorectal cancer (CRC) is the third most commonly diagnosed cancer worldwide, with increasing incidence rates in emerging economies [9]. Given these alarming statistics, there is an urgent need for innovative approaches to prevent and treat this disease. One promising strategy involves inducing programmed cell death (apoptosis) in cancer cells, which can inhibit uncontrolled cell proliferation and tumor progression [10]. In this context, naturally derived compounds, including bioactive proteins and peptides, have gained considerable attention for their antiproliferative and proapoptotic properties [11]. Our study aimed to evaluate the antiproliferative and proapoptotic activity of quinoa proteins and their tryptic hydrolysates (i.e., peptides) using in vitro functional assays such as 3‐(4,5‐dimethylthiazol‐2‐yl)‐2,5‐diphenyltetrazolium bromide (MTT) for cytotoxicity and proliferation. Concentration–response curves for proteins and peptides revealed that certain quinoa varieties significantly reduced cell growth at certain concentrations. To further investigate the underlying mechanisms, we employed data mining to identify cytotoxic proteins in the comprehensive protein maps of each quinoa variety established by LC‐MS/MS shotgun proteomics. Multivariate data analysis allowed discrimination of quinoa varieties based on the relative abundance of their cytotoxic proteins in relation to the bioactivity observed in functional assays. Additionally, the differential expression of apoptosis‐related biomarkers in Caco‐2 cells was studied by targeted LC‐MS/MS proteomics, providing evidence for a potential link between quinoa‐derived cytotoxic proteins and apoptotic pathways in CRC cells, as well as identifying potential bioactive candidates. This comprehensive approach, including functional assays, LC‐MS/MS proteomics, data mining, and multivariate data analysis, aims to evaluate the in vitro biological effect and provide initial mechanistic insights into the apoptosis‐related actions of quinoa proteins and hydrolysates derived from quinoa grains. Ultimately, the purpose of our study is to highlight the multifaceted potential of quinoa grains in the broader field of functional foods and nutraceuticals.

## 2 Materials and Methods

### 2.1 Chemicals

Ammonium bicarbonate (ABC ≥ 99.5%), acetonitrile (HPLC purity), methanol (HPLC purity), formic acid (analytical reagent grade), and urea were purchased from Carlo Erba (Milan, Italy). Boric acid (≥99.5%) was supplied by Merck (Darmstadt, Germany). Protein assay dye reagent concentrate was purchased from Bio‐Rad (Hercules, CA, USA). Bovine serum albumin (BSA), trypsin from bovine pancreas, iodoacetamide (IAA), dithiothreitol (DTT), sodium carbonate, Folin–Ciocalteu (FC) reagent, hydrochloric acid (HCl, 37% (v/v)), and sodium hydroxide (NaOH, ≥ 98%) were purchased from Sigma–Aldrich (Darmstadt, Germany). Bicinchoninic acid (BCA) protein assay kit was purchased from ThermoFisher Scientific (San José, CA, USA). Deionized ultrapure water (herein referred as water) was purified (0.055 µS/cm, TOC 1 ppb) with a Purelab pulse + Flex ultra‐pure water system (Elga Veolia, Milan, Italy).

### 2.2 Plant Materials

B, R, W, and RO quinoa grains were acquired from local supermarkets in Barcelona (Spain). The contents of the 500 g commercial packages (3 packages per commercial grain variety) were homogenized, dried in an air‐current oven at 40°C for 24 h, ground in a coffee grinder, and stored at room temperature in a desiccator.

### 2.3 Cell Culture

Human colorectal adenocarcinoma cells Caco‐2 (ATCC HTB37, American Type Culture Collection, Manassas, VA, USA) were grown in Dulbecco's Modified Eagle's Medium (DMEM) high glucose cell media (Euroclone spa, Milan, Italy) supplemented with 10% fetal bovine serum (FBS, Euroclone spa), 1% of penicillin–streptomycin (Gibco, NY, USA), and 1% of nonessential amino acid solution (Gibco, Paisley, UK). The cell line was grown in a humidified atmosphere of 95% air and 5% CO_2_ at 37°C.

### 2.4 Extraction and Sample Preparation of Proteins and Peptides

Quinoa proteins were extracted as described by Galindo‐Luján et al. [6, 12]. Two hundred and fifty milligram of the ground sample were mixed with 1 mL of water and 39 µL of 1 mol/L NaOH (final pH value of 10.0) using a vortex Genius 3 (Ika, Staufen, Germany). The suspension was incubated for 1 h at 36°C with constant shaking at 900 rpm in a TS‐100 thermoshaker (Biosan, Riga, Latvian Republic). Separation of soluble proteins from the insoluble residue was performed by centrifugation at 15 000 × *g* for 20 min at 4°C in a cooled Rotanta 460 centrifuge (Hettich Zentrifugen, Tuttlingen, Germany). For protein purification, the supernatant pH was adjusted with 22 µL of 1 mol/L HCl to a final pH value of 5.0. After centrifugation at 15 000 × *g* for 20 min at 4°C, precipitated proteins were resuspended in 1 mL of a sodium borate buffer (60 mmol/L H_3_BO_3_ adjusted to pH 9.0 with NaOH). The mixture was incubated at 36°C for 1 h with constant shaking at 1200 rpm to aid solubilization, and the solution was filtered through a 0.22 µm nylon filter (Macherey‐Nagel, Düren, Germany). Protein extracts were evaporated to dryness using a Savant SPD‐111 V SpeedVac concentrator (ThermoFisher Scientific) and stored at −20°C until use. To facilitate protein solubilization and tryptic digestion, dried protein extracts were reconstituted in a solution of urea 3 mol/L in 50 mmol/L ABC (final pH value of 8) for 20 h at room temperature with constant shaking at 900 rpm. Subsequently, protein extracts were centrifuged at 15 000 × *g* for 10 min at 4°C, and the supernatants were concentrated and desalted with a buffer exchange centrifugal filtration procedure using 50 mmol/L ABC (Amicon Ultra‐0.5 cellulose acetate centrifugal filters; 3 kDa molecular weight cutoff, Millipore, Bedford, MA, USA). Briefly, 500 µL of the sample solution was centrifuged at 10 000 × *g* for 10 min at 25°C, and the residue was washed with 50 µL of ABC 50 mmol/L five times under the same conditions. Finally, water was used to perform two additional steps of washing and desalting. The obtained residue was recovered by inverting in a new vial the upper reservoir and spinning at a reduced centrifugal force (300 × *g* for 2 min). Then, 50 mmol/L ABC was added to adjust the final volume to 500 µL. Purified protein extract solutions were assayed for total protein content according to the Bradford method [13]. Twenty microliters of each extract solution or BSA standard solution was added to 1000 µL of previously diluted (1:5 v/v) Bradford reagent, and absorbance was measured at 595 nm. BSA standard solutions were prepared by serial dilution to cover the 25–400 µg/mL concentration range from a 1 mg/mL BSA stock solution. Protein hydrolysates were obtained after tryptic digestion of the purified protein extract solutions. Proteins were reduced with DTT (100 mmol/L for 30 min at 37°C), followed by alkylation with IAA (200 mmol/L for 30 min in the dark), and again the addition of DTT (100 mmol/L for 15 min at room temperature) to avoid overalkylation. Finally, samples were digested by incubation with trypsin (1:50 m/m enzyme/substrate ratio) at 37°C overnight with constant shaking at 1200 rpm. The digestion was stopped by the addition of formic acid 1% (v/v). The supernatant with the tryptic peptides was collected after centrifuging at 15 000 × *g* for 2 min at 4°C. Peptide hydrolysates were dried under nitrogen and stored at −20°C until use.

### 2.5 MTT Cytotoxicity and Proliferation Assay

The cytotoxicity of quinoa grain proteins and peptides on Caco‐2 cells was evaluated at 24 and 72 h, respectively. To provide a wide overview of the biological effect, a concentration–response curve was constructed including four different concentrations. Proteins and peptides were assayed in duplicate at 4, 2, 1, and 0.5 mg/mL. To avoid any external interference, such as interaction between the extraction buffer and cell culture, the highest concentration for each dried extract was reconstituted using cell media (DMEM high glucose). This reconstitution was performed inside a biological safety cabinet, without any additional sources of proteins (FCS, BSA), to mitigate the risk of microbial contamination. Preliminary trials were conducted to assess the solubility of the extracts in cell media and to achieve a final solution pH within the range of 7–7.4. Caco‐2 cells were seeded in 96‐well plates at a density of 5·10^4^/0.2 mL, in complete medium until reaching ∼80% confluence, and were serum‐starved overnight. The following day, cells were treated with the extracts (100 µL/well) according to the concentration–response design, where 10% FCS and 10% dimethylsulfoxide (DMSO) served as negative and positive controls for cell viability, respectively. Cell viability was evaluated after 24 h of treatment at 37°C and 5% of CO_2_, using the MTT colorimetric assay [14]. MTT was added at a final concentration of 1 mg/mL and incubated for 2 h at 37°C. The resulting formazan crystals were solubilized with 100 µL of DMSO per well. Absorbance was measured at 570 nm using a TECAN Spark 10 M spectrophotometer (Männedorf, Switzerland). To evaluate the impact of quinoa proteins and peptides on cellular proliferation, the same protocol was followed, but with a 72‐h exposure time [15]. The extract concentrations that reduced cell viability by more than 20% from the basal condition (cells treated with medium only, corresponding to 100% viability) were excluded from further experiments. Conversely, concentrations that were nontoxic at 24 h but induced a reduction in cell viability at 72 h underwent a second proliferation assay to elucidate the molecular mechanisms underlying this in vitro biological effect. Concentration–response curves were made, averaging the viability results obtained from two biological replicates (*n* = 2).

MTT assay data were analyzed using a one‐way ANOVA statistical test with Prism statistical analysis package version 6 (GraphPad Software, Boston, MA, USA). Results were plotted as mean ± standard deviation (SD). A *p* value ≤ 0.05 was considered statistically significant. Cell viability was normalized against the basal condition (cell growth in cellular media).

### 2.6 Sample Preparation for Functional Assays and LC‐MS/MS Proteomics Antiproliferative Insight

The concentrations of quinoa protein extract that showed potential antiproliferative activity were exposed to Caco‐2 for 72 h following the previous procedure. Cells treated with 4 mg/mL BSA and 10% FCS were used as a negative control to assess cell viability in vitro. This step was performed to ensure that the cells were functional and viable before proceeding with the targeted proteomic analysis of apoptosis biomarkers. For this analysis, cells grown in basal medium conditions (basal medium without any additional protein source) were used as a negative control. Conversely, paclitaxel (taxol) was used as a positive control due to its known apoptosis‐inducing effect [16]. Caco‐2 cells were seeded in 12‐well plates at an elevated cellular density (5·10^5^/mL) and exposed to treatments (350 µL). After 72 h (at 37°C with 5% of CO_2_), the cell media containing extracts and controls was removed, and the cell monolayer was washed with phosphate‐buffered saline (PBS). To recover cellular debris present in the washing solution, cells were centrifuged at 15 000 × *g* for 10 min at 25°C, and the cellular pellet was isolated. For the collection of cellular proteins, the entire pool of Caco‐2 cells (monolayer + pellet) was lysed using an aqueous solution of NaOH 0.1 mol/L overnight at 4°C with gentle stirring. The protein content of the lysate was determined using a BCA assay kit (*λ* = 550 nm) [17, 18], calibrating with BSA protein standard in the 2.5–100 µg protein content range. The protein lysate was then dried under nitrogen and reconstituted in 50 mmol/L ABC (pH = 8) to recreate the optimal environment for tryptic digestion. Proteins were digested following the aforementioned tryptic digestion procedure. The resulting peptides were dried under nitrogen and reconstituted in 50 µL of a water/acetonitrile/formic acid aqueous solution (49.95/49.95/0.1, v/v/v) for LC‐MS/MS analysis.

### 2.7 Targeted LC‐MS/MS Proteomics

Peptide separation of the tryptic digests was carried out by using an Xbridge Peptide BEH C18 (250 mm total length (*L*_T_) × 2.1 mm internal diameter (i.d.), 5 µm particle size) column (Waters, Milford, MA, USA) equipped with a C18 (5 mm *L*_T_ × 2.1 mm i.d.) prefiltering column (Waters). Mobile phases, consisting of Solvent A (0.1% v/v aqueous formic acid) and Solvent B (0.1% v/v formic acid in acetonitrile), were delivered in gradient elution at a flow rate of 200 µL/min. The linear gradient was programmed as follows: 0 min 2% Solvent B, 4 min 2% Solvent B, 150 min 90% Solvent B, 155 min 90% Solvent B, and 160 min 2% Solvent B. Ten microliters of the sample was injected. All targeted LC‐MS/MS analyses were carried out using an Agilent Technologies 1200 series HPLC (Agilent Technologies, Waldronn, Germany) coupled with a pneumatically assisted electrospray ionization (ESI) interface to a QTRAP 4000 triple quadrupole mass spectrometer (AB SCIEX, Foster City, CA, USA). The ESI voltage and capillary temperature were set at 5.5 kV and 350°C, respectively, with sheath and auxiliary gases delivered at flow rates of 45 and 5 (arbitrary units). Experiments were conducted under positive ion‐single reaction monitoring (SRM) conditions using nitrogen as a collision gas (pressure of 2.1  ×  10^−3^ mbar) and a 20 ms dual time for each monitored transition. Skyline software (MacCoss Lab Software, version 22.2.0.351) was employed to simulate the tryptic digestion of 13 protein biomarkers for the LC‐MS/MS targeted proteomic analysis. MS/MS transitions (containing 3*y* and 3*b* fragments for each peptide) were analyzed in double (*n* = 2) to confirm peptide identity based on fragments coelution and retention time consistency between replicates; only proteins found in both replicates were considered for the statistical analysis. Collision energy values were predicted using simple linear models [19], and data were statistically analyzed using MSstats, an R‐based environment that allows multiple data comparison for quantitative bottom‐up mass spectrometry‐based proteomic experiments to detect differentially abundant proteins [20]. Declustering potential was set to 80 V, and the collision energy was optimized according to the peptide sequences. Details of the protein biomarkers, target peptides, SRM transitions, and collision energies are provided in Table S7. Each tryptic digest sample was analyzed in duplicate, and the analytes were relatively quantified considering their relative peak areas and normalizing against the total protein content measured by the BCA assay.

### 2.8 Data Analysis and Interpretation

#### 2.8.1 Identification of Cytotoxic Proteins in Quinoa Grain Extracts

In a previous study, we described a proteomics data mining strategy to identify quinoa grain proteins with potential immunonutritional activities [7], which was adapted here to uncover potential cytotoxic proteins. FASTA sequences from plant‐derived peptides and proteins known for their cytotoxic activities were searched and retrieved from the National Center for Biotechnology Information (NCBI) database (https://www.ncbi.nlm.nih.gov/protein, accessed on July 1, 2023). The cytotoxic protein types that contained certain protein sequences with a percent identity ≥20% with the experimental proteome map of quinoa grains [6, 7], included ribosome‐inactivating proteins (RIPs, 2877 proteins from different plant species), protease inhibitors (PIs, 238 proteins from *Arabidopsis thaliana*), α‐amylase inhibitors (AIs, 670 proteins from *A. thaliana*), pore‐forming toxins (PFTs, 637 proteins from different plant species), and antimicrobial peptides (AMPs, 675 proteins from different plant species [7]). As can be observed, in the most general cytotoxic protein types, the search was filtered by plant organism, selecting the model plant organism *A. thaliana*, to avoid information overload. Several other proteins with cytotoxic activity were also considered in the preliminary search, but no significant similarities with the experimental proteome map of quinoa grains were found. The discarded cytotoxic protein types included beta‐purothionins (40 proteins from different plant species), ureases (208 proteins from different plant species), and arcelins (275 proteins from *A. thaliana*). Protein entries for selected RIPs, PIs, AIs, PFTs, and AMPs can be found in Tables S1–S5, respectively. To assess sequence similarity, the obtained FASTA sequences from plant‐derived cytotoxic peptides and proteins were compared, as described in our previous study, to the Reference Sequence (RefSeq) NCBI quinoa database (63 373 protein entries) using the protein–protein Basic Local Alignment Search Tool (BLASTp) of the NCBI (https://blast.ncbi.nlm.nih.gov/Blast.cgi?PROGRAM = blastp&PAGE_TYPE = BlastSearch&LINK_LOC = blasthome, accessed on July 1, 2023). The BLASTp tool enables the detection of multiple local alignments between two protein sequences and supplies information for internal sequence matches. It is noteworthy that, for each quinoa protein, only matches with the cytotoxic sequences providing the highest percent identity were selected. Subsequently, NCBI entries corresponding to quinoa proteins with potential cytotoxic bioactivities were searched against the experimental quinoa grain proteome map from R, B, W, and RO quinoa grains obtained by LC‐MS‐MS shotgun proteomics in our previous work [6] (1211 proteins). The average normalized label‐free (LFQ) intensities of the potential cytotoxic proteins identified in R, B, W, and RO quinoa grains (*n* = 2) (Table S6) were considered for principal component analysis (PCA) and PLS‐DA. PCA allowed the unsupervised identification of trends and clustering of the data, as well as the detection of outliers. PLS‐DA was then applied to build a classification model with improved class separation and to reveal the importance of the different protein biomarkers for discrimination, taking into account their variable importance in the projection (VIP) scores (VIPs > 1 were considered relevant for the models) [12]. A leave‐one‐out cross‐validation of the PLS‐DA model was performed during model optimization, and “mdatools” R package (version 0.12.0) was used for PCA and PLS‐DA [21].

#### 2.8.2 Differential Expression of Apoptosis Biomarkers in Caco‐2 Cells

Apoptosis‐related molecular biomarkers and their biological networks are well described in the literature [22, 23, 24]. Starting from these valuable insights, the selection of the apoptosis protein biomarkers to investigate was made using Cytoscape (version 3.10.1, Cytoscape Consortium, https://cytoscape.org) pathway enrichment analysis (g:SCS as the default method for computing multiple testing correction), inspecting the human apoptosis pathway (WP254) (Table S7). The MS raw files were processed with Skyline for peptide and protein identification and relative quantification. Peptide MS/MS transitions were obtained, and search parameters included cysteine carbamidomethylation as a fixed modification, and N‐terminal acetylation and methionine oxidation were designated as variable modifications. Up to two missed cleavages were allowed for the tryptic digestion. The uniqueness of candidate peptide sequences was assessed using the BLASTp tool. MSstats (integrated within Skyline) was used for a group comparison with a 95% confidence level (Benjamini–Hochberg algorithm was applied to attain the adjusted *p* values), considering relative protein abundance values to pairwise condition comparisons. RStudio (version 4.4.1) packages such as “ggplo2” and “gplots” were used to create heatmaps (row‐scaled). LC‐MS/MS peak area and percent Relative Standard Deviation (RSD) values for the apoptosis biomarkers and statistical significance (adjusted *p* value) of RO and R quinoa peptides against negative (basal) and positive (taxol) controls are reported in Tables S8 and S9, respectively. To evaluate a possible correlation between the most relevant cytotoxic proteins identified through data mining in R and RO quinoa grains and the targeted proteomics LC‐MS/MS peak areas of significantly differentially expressed (at least compared to the negative control) apoptosis biomarkers triggered by R/RO peptides, a PCA and a Pearson correlation matrix were calculated from the row‐scaled data.

## 3 Results and Discussion

### 3.1 Cytotoxic and Antiproliferative Potential of Proteins From Different Quinoa Grain Varieties

In this study, we aimed to investigate whether proteins or protein hydrolysates (i.e., peptides) from different commercial quinoa varieties exhibit antiproliferative effects on colorectal adenocarcinoma cells. Our rationale was to establish a correlation between the protein profiles of quinoa grain extracts and their impact on cellular activity. This was achieved through an integrated approach that combined experimental evidence from functional assays with LC‐MS/MS shotgun and targeted proteomics. Using data mining and multivariate data analysis, we identified putative bioactive proteins within the extracts that may be responsible for the observed biological effects. Additionally, we analyzed the differential expression of apoptosis biomarkers in Caco‐2 cells, providing an initial mechanistic explanation for the observed biological action.

#### 3.1.1 Cytotoxicity and Proliferation Functional Assays

**FIGURE 1:**
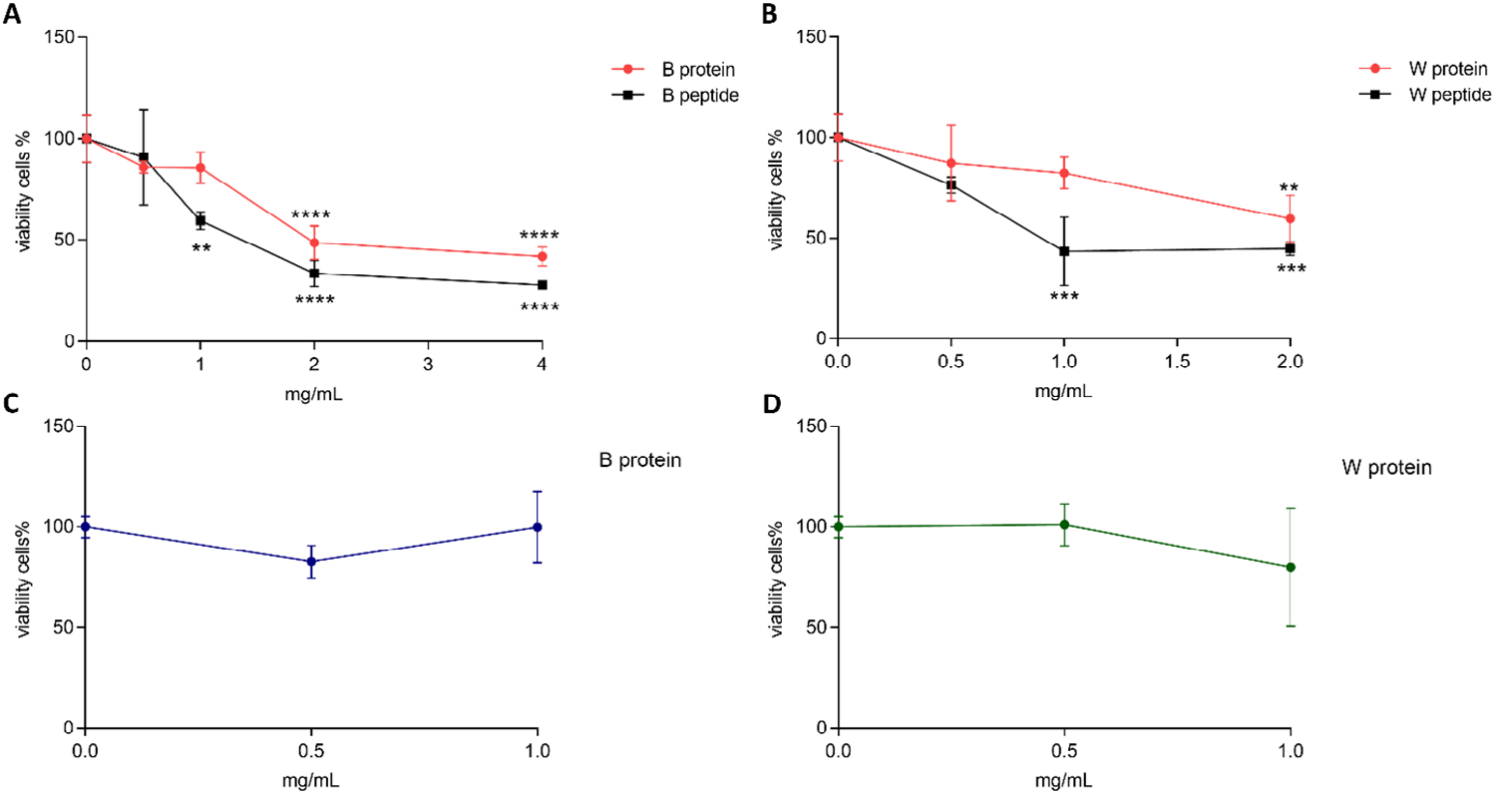
(A) Cytotoxicity (24 h) assay concentration–response curve for B quinoa proteins and peptides. (B) Cytotoxicity (24 h) assay concentration–response curve for W quinoa proteins and peptides. (C) Proliferation (72 h) assay performed on noncytotoxic concentrations of B proteins. (D) Proliferation (72 h) assay performed on noncytotoxic concentrations of W proteins. Data are reported as mean ± standard deviation (SD) (*n* = 2). *p* values ≤ 0.05 (*), ≤0.01 (**), ≤0.001 (***), and ≤0.0001 (****) were considered statistically significant compared to the control (cells treated with basal medium only).

**FIGURE 2:**
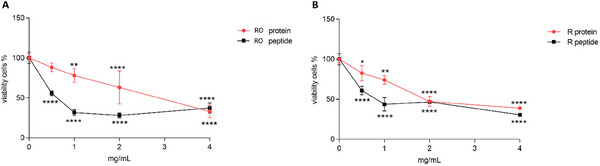
(A, B) Cytotoxicity (24 h) assay concentration–response curve for Royal White (RO) and Red (R) quinoa proteins and peptides. Given their cytotoxicity, none of them were tested for cellular proliferation. Data are reported as mean ± standard deviation (SD) (*n* = 2). *p* values ≤ 0.05 (*), ≤0.01 (**), ≤0.001 (***), and ≤0.0001 (****) were considered statistically significant compared to the control (cells treated with basal medium only).

In the context of pharmacology and toxicology [25], the MTT assay was used to generate concentration/dose–response curves to assess the cytotoxicity at 24 h of quinoa grain proteins and peptides. Additionally, the reduction in cell viability at 72 h was considered to evaluate a possible antiproliferative effect. Cell viability was assessed using the MTT assay. To ease visual representations of results, quinoa varieties with similar activities were grouped in Figures 1 and 2. Figure 1 shows the cytotoxicity and proliferation concentration–response curves for B and W quinoa proteins and peptides. As can be observed in Figure 1A, B, B and W quinoa proteins showed a clear cytotoxic effect at 4 and 2 mg/mL, whereas lower concentrations (0.5 and 1 mg/mL) showed no significant cytotoxicity. These lower concentrations were retested for effects on cellular proliferation at 72 h (Figure 1C, D), confirming their safety. In contrast, peptides were able to reduce cell viability even at low concentrations, with a well‐defined concentration–response curve (Figure 1 A, B). Among these, 0.5 mg/mL was the only concentration that demonstrated a slight, nonsignificant inhibition of cell growth. After confirming their safety at 72 h (data not shown), B and W peptides at 0.5 mg/mL were promoted to the apoptosis investigation step.

Figure 2 shows the functional assay results for R and RO proteins and peptides. As can be observed, a remarkable concentration‐dependent toxic trend was evident at 24 h for both RO and R quinoa proteins and peptides, starting at a concentration of 0.5 mg/mL. Notably, the cellular effects of peptides appeared stronger compared to proteins. The differences in bioactivity between proteins and peptides are supported by existing experimental evidence, where peptides have been preferred in therapeutic applications, such as antimicrobial and anticancer treatments [26, 27]. Peptides can exhibit toxicity against cancer cells due to several chemical properties and mechanisms. However, it is important to note that the toxicity can vary significantly depending on the specific peptide sequence and the type of cancer cells being targeted [28]. Peptides may be involved in multiple anticancer mechanisms, depending on their specific structures, including disruption of cellular function (such as DNA replication, RNA transcription, and protein expression), membrane penetration, immune activation, and induction of apoptosis [28]. Based on our findings, a concentration of 0.5 mg/mL was selected to evaluate the antiproliferative effect of R and RO quinoa proteins, while a concentration of 0.1 mg/mL was chosen for their peptides. Although this lower concentration was not assessed using the MTT test, it was reasonable to select a significantly lower concentration than 0.5 mg/mL due to the high toxicity of peptides.

#### 3.1.2 Identification of Cytotoxic Proteins

**FIGURE 3:**
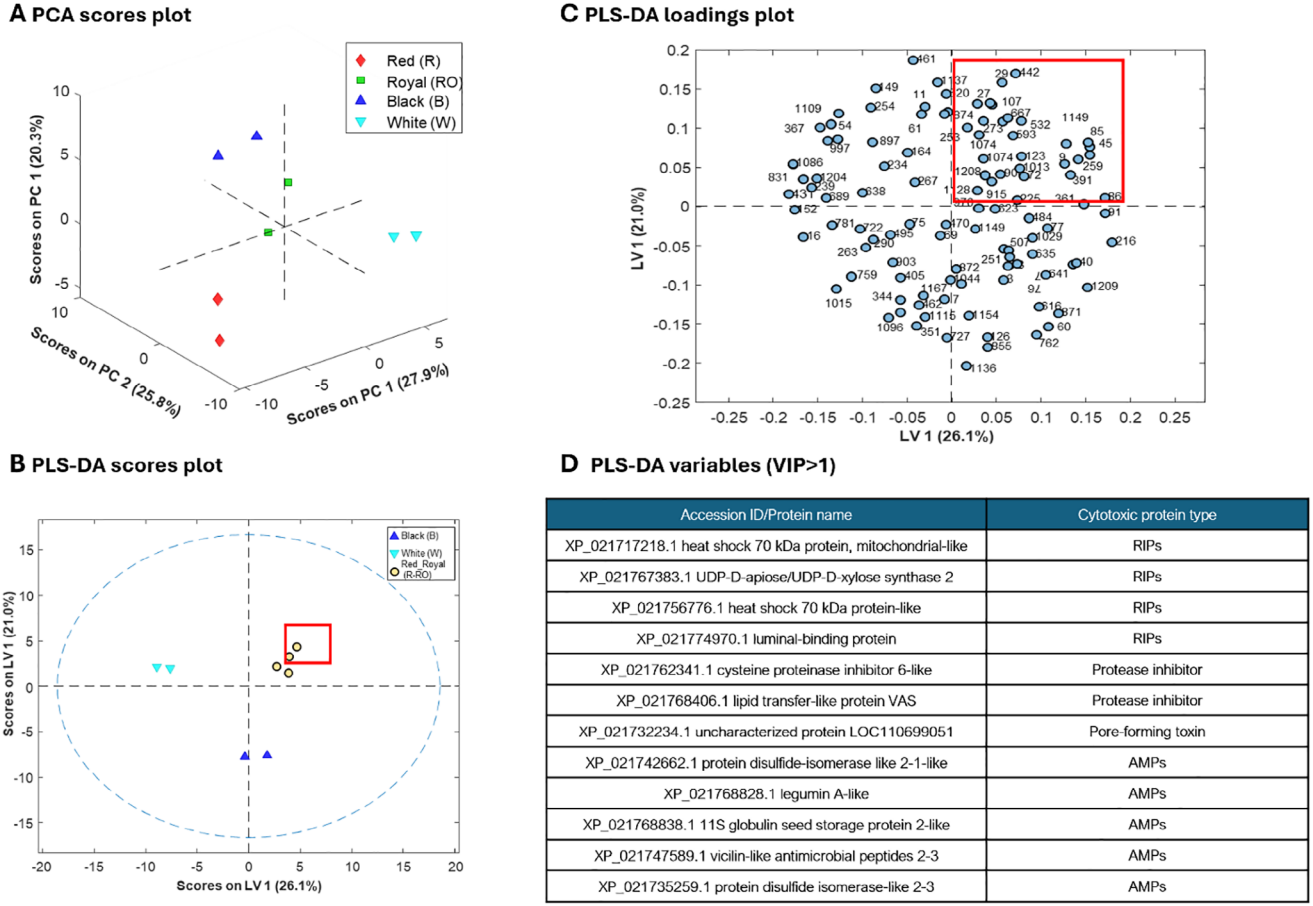
Principal component analysis (PCA) and PLS‐DA classification model results for the different quinoa protein extracts (average normalized label‐free [LFQ] intensities of the 107 cytotoxic proteins identified were used as variables, four quinoa varieties, and two independent extracts). (A) PCA scores plot of a three principal component (PC) model. (B) PLS‐DA scores plot of a two latent variable (LV) model (R and RO quinoa were grouped as a single class according to the functional assays). (C) PLS‐DA loadings plot, and (D) list of cytotoxic proteins with VIP scores greater than 1 and positive LV1 and LV2. The protein IDs are shown in Table S6.

Data mining for proteins and peptides with toxic activity on tumor cells involves employing advanced computational techniques to analyze extensive biological data repositories. This approach serves to identify specific proteins and peptides capable of selectively targeting and damaging cancerous cells [29]. Although this process provides valuable insights into potential anticancer agents and their molecular mechanisms, deciphering the molecular interplay can present significant challenges [30]. In recent studies, we employed label‐free LC‐MS/MS shotgun proteomics to generate comprehensive proteome maps of quinoa grains from four commercial quinoa varieties (R, B, W, and RO) [6]. These maps were further analyzed to identify proteins with potential immunonutritional bioactivities [7]. Using these experimental proteome maps, a similar data mining strategy was applied in the current study to identify proteins with potential cytotoxic activity in the same quinoa varieties. In particular, five types of cytotoxic proteins were considered to construct a specific database of cytotoxic plant proteins: RIPs, PIs, AIs, PFTs, and AMPs. This effort was constrained by the limited literature and database information available on cytotoxic proteins in quinoa [31], relying primarily on data from other plants, including the model organism *A. thaliana*. The resulting database, containing 5097 protein entries, was screened against the experimental proteome maps of R, B, W, and RO quinoa grains [6]. Following this procedure, a total of 107 quinoa grain proteins were identified across the four quinoa varieties as potentially cytotoxic. Detailed information about the identified proteins can be found in Table S6. From this comprehensive in‐silico characterization and considering the relative abundances of these proteins in the different quinoa grain varieties (i.e., their average normalized LFQ intensities), a multivariate data analysis workflow was developed to investigate variations among the quinoa varieties. First, PCA was performed to evaluate the multivariate profile, aiming to identify possible clusters, patterns, and outliers. Subsequently, PLS‐DA was applied to build a refined classification model able to find a correlation between the potential cytotoxicity of the quinoa proteins and the cell‐growth reduction observed in the functional assays. The results from both PCA and PLS‐DA are presented in Figure 3.

The PCA scores plot (Figure 3A) revealed that the four quinoa grain varieties had distinct profiles of cytotoxic proteins, allowing their separation based on three principal components. This separation laid the foundation for building the PLS‐DA classification model. The PLS‐DA model was established grouping R and RO quinoa samples as a single class (R‐RO), based on their proximity in the PCA scores plot (Figure 3A). This grouping aligned with their remarkable concentration‐dependent cytotoxicity in the functional assays. As can be observed in the PLS‐DA scores plot (Figure 3B), the model effectively classified quinoa samples into the three considered classes. The PLS‐DA loadings plot (Figure 3C) allowed the identification of cytotoxic proteins that were particularly characteristic of each class. See, for example, for the R‐RO quinoa class samples, the cytotoxic proteins highlighted by a square in the loading plot (LV1 and LV2 > 0, as in the scores plot). To verify if these proteins were relevant to explain the excellent classification of R‐RO quinoa samples, VIP scores of the cytotoxic proteins were calculated considering the separation of B, W, and R‐RO quinoa samples from the rest of the classes. Figure 3D summarizes the list of cytotoxic proteins with VIP scores greater than 1, which were significantly meaningful for the classification, and positive LV1 and LV2. Among these 11 proteins, three were RIPs, two PIs, one PFT, and five AMPs. These proteins are considered the most relevant for explaining the discrimination between the most cytotoxic R‐RO quinoa class and the other two classes, as they exhibit notably higher abundance in these two quinoa varieties (LV1 and LV2 > 0 and Table S6) and significant VIP scores.

Overall, the results from data mining and multivariate data analysis supported the findings from the functional assays. Given the diverse cellular effects exhibited by the most discriminating cytotoxic proteins and their potential association with antiproliferative effects, we decided to explore the apoptosis pathway to better understand the underlying molecular mechanisms.

### 3.2 Proapoptotic Insights Into the Potential Antiproliferative Activity of Proteins From Different Quinoa Grain Varieties

#### 3.2.1 Proliferation Functional Assay and Targeted LC‐MS/MS Proteomics

**FIGURE 4:**
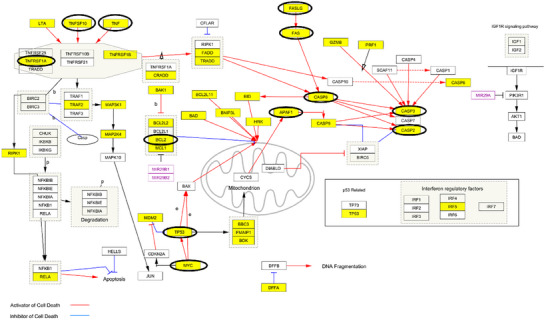
Schematic representation of the human apoptosis network (Cytoscape Consortium). Highlighted genes (in yellow) represent the results of an expression enrichment analysis focused on the regulation of the apoptosis pathway. Twelve of the thirteen investigated biomarkers are highlighted with black circles, namely tumor necrosis factor α (TNFA), tumor necrosis factor receptor 1A (TNR1A), tumor necrosis factor receptor 6 (FAS), its ligand (FASL), caspase 2,3,8 (CASP 2‐3‐8), apoptotic protease activating factor‐1 (APAF1), proto‐oncogene protein (MYC), B‐cell lymphoma 2 (BCL‐2), cellular tumor antigen (p53), and tumor necrosis factor‐related apoptosis‐inducing ligand 10 (TRAIL). Only the expression of the tumor necrosis factor receptor 10C (TNFRSF10C) apoptosis biomarker was not revealed through gene expression enrichment analysis (not shown in the Figure). Red arrows depict activators of cell death, while blue arrows stand for inhibitors of cell death.

To investigate a potential antiproliferative effect explaining the observed reduction in cell viability during proliferation assays, selected concentrations of quinoa grain proteins and peptides were tested under different experimental conditions. After 72 h of exposure, cells were lysed, and cellular proteins were collected for analysis. LC‐MS/MS targeted proteomics was then employed to measure the peak areas of selected apoptosis biomarkers (Table S8). For comparison, cells treated with basal medium or 10 nmol/L paclitaxel served as negative and positive controls, respectively. To account for inherent variability among cell lines, alternative protein sources, including FCS (10%) and BSA (4 mg/mL), were evaluated to rule out any potential cell growth inhibition caused by the presence of proteins, regardless of their source. LC‐MS/MS analyses under these conditions showed no differential expression compared to the basal medium. Regarding the apoptosis biomarkers (Table S7), they were selected considering the human apoptosis pathway (Cytoscape Consortium) and a gene expression enrichment analysis focusing on apoptosis regulation. Programmed cell death and antiproliferative effects are interconnected, as inhibiting cell proliferation can lead to apoptosis [32]. Halting the rapid division of cancer cells can push them into a state more susceptible to apoptosis. Figure 4 illustrates the resulting human apoptosis network, highlighting genes associated with the regulation of the apoptosis pathway and apoptosis biomarkers. The selection of apoptosis biomarkers was further supported by evidence from the literature on the differential expression induced by tumor antiproliferative molecules, such as paclitaxel [33, 34, 35, 36]. Specifically, 13 apoptosis biomarkers were investigated, 12 of which are highlighted with circles in Figure 4. Notably, only one biomarker was not identified through the gene expression enrichment analysis (i.e., tumor necrosis factor receptor 10C [TNFRSF10C]). The relative abundances of these 13 apoptosis biomarkers were satisfactorily determined by measuring their peak areas by LC‐MS/MS (RSD ≤ 15% (*n* = 2), Table S8).

#### 3.2.2 Differential Expression of Apoptosis Biomarkers

**FIGURE 5:**
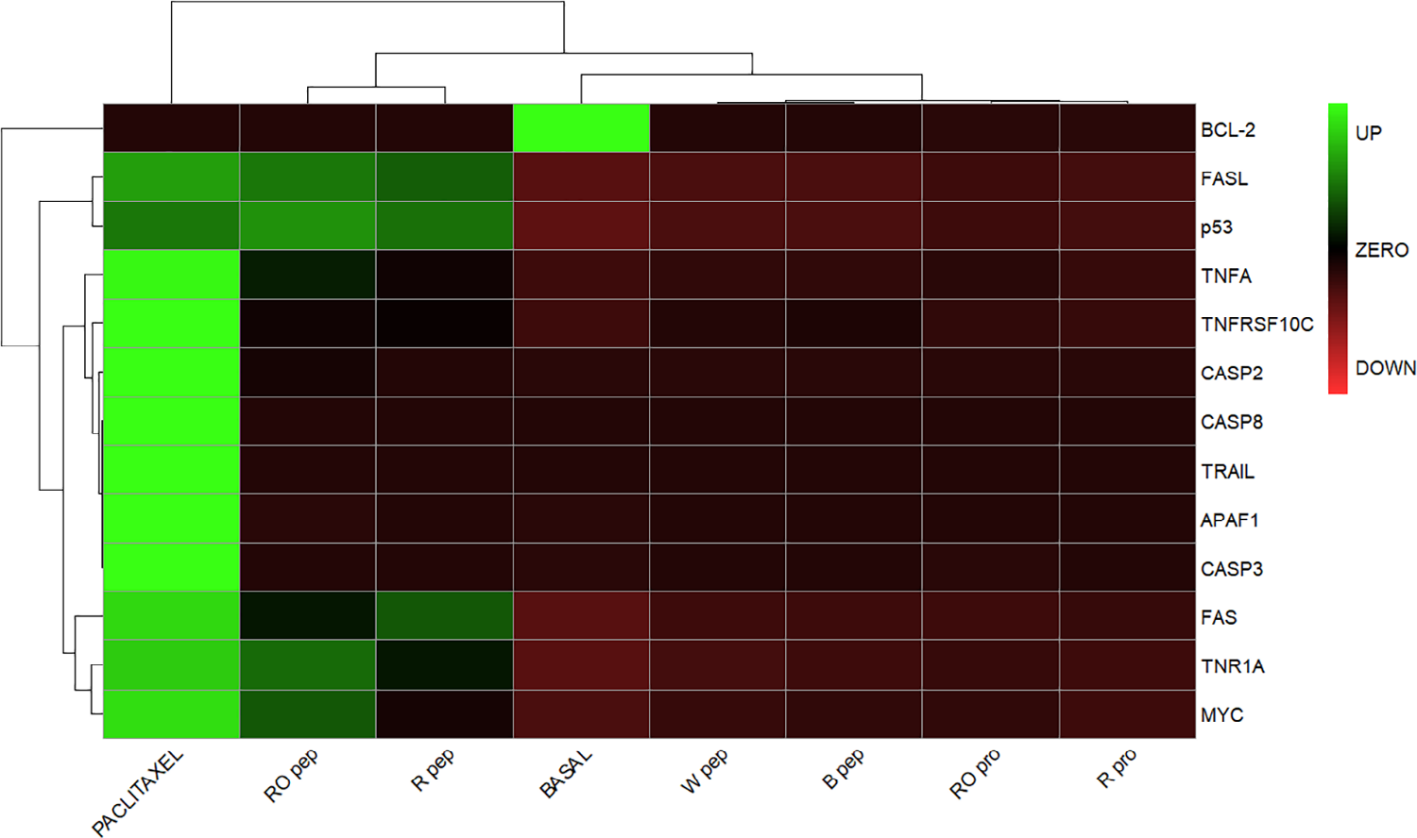
Heatmap of differentially expressed apoptosis biomarkers for the negative control (basal), positive control (10 nmol/L paclitaxel), 0.5 mg/mL White (W)/Black (B) peptides (pep), 0.5 mg/mL Red (R)/Royal White (RO) proteins (pro), and 0.1 mg/mL R/RO peptides (pep). The relative abundances of the detected peptides for each protein biomarker were used in the calculations. Green, red, and black boxes represent upregulated, downregulated, and unchanged protein expression, respectively.

The relative abundance of apoptosis protein biomarkers was compared across the different conditions: the negative control for proliferation (basal medium), the positive control (taxol), 0.5 mg/mL R/RO proteins, 0.1 mg/mL R/RO peptides, and 0.5 mg/mL B and W peptides. Pairwise condition comparisons were conducted using a linear mixed‐effects model based on the Student's *t* distribution [20]. Initially, the protein expression profiles associated with quinoa grain proteins and peptides were compared to the negative control of proliferation (basal medium) to identify statistically significant upregulation of cell death activators or downregulation of inhibitors. This analysis excluded an antiproliferative mechanism triggered by B and W peptides, supporting their safety at 24 h, as evaluated using the MTT assay (Figure 1A, B). Similarly, none of the differential expressions induced by R, and RO proteins were statistically significant in the pairwise comparisons against the negative control (*p* value > 0.05). The slight cytotoxicity observed at 24 h under these conditions could potentially be explained by alternative mechanisms, as suggested by data mining of quinoa grain proteins with cytotoxic properties (i.e., RIPs, PIs, PFT, and AMPs). In contrast, statistically significant differential expressions were observed for the R and RO peptides. To further investigate the apoptosis pathway, the relative abundances of apoptosis biomarkers for all the conditions were used to construct a heatmap, as shown in Figure 5. This comparison identified five distinct clades corresponding to taxol, basal medium, B/W peptides, R/RO proteins, and R/RO peptides.

As can be observed in Figure 5, the samples corresponding to R/RO proteins and B/W peptides were grouped under the same upper clade and, in turn, with basal condition, as they were not able to trigger any apoptosis‐related effects. In contrast, R/RO peptides formed a separate cluster. The observed upregulation of some key players in the apoptosis network suggested a cellular response resembling the one induced by paclitaxel. However, it is difficult to compare the cellular effects induced by a complex mixture of peptides with those of a pure compound such as paclitaxel, a diterpene originally isolated from the bark of *Taxus brevifolia* (Pacific yew) [37] and widely used as a chemotherapy drug with a well‐documented potency [38]. Anyway, pathway analysis was conducted to find out a reasonable connection between the functional and the targeted proteomics results.

We started by analyzing the paclitaxel‐induced antiproliferative profile. Several tumor necrosis factors and receptors were found upregulated in cells treated with paclitaxel, including tumor necrosis factor α (TNFA), tumor necrosis factor receptor 1A (TNR1A), tumor necrosis factor receptor 6 (FAS), and its ligand (FASL). Additionally, certain downstream products of the TNF signaling cascade, such as caspase 2‐3‐8, were significantly upregulated (*p* value < 0.01 in all cases). Supporting the contribution of caspases, the apoptotic protease activating factor‐1 (APAF1) was also highly upregulated in paclitaxel‐treated cells (*p* value < 0.001). APAF1 is a key molecule in the intrinsic (mitochondrial) apoptosis pathway, contributing to the molecular assembly of the apoptosome, a large multiprotein complex. APAF1 mediates the cytochrome c‐dependent autocatalytic activation of procaspase‐9 (APAF3), an initiator caspase recruited and activated by the apoptosome. This activation leads to downstream caspase 3 processing [39]. Furthermore, an equilibrium between the expression of MYC proto‐oncogene protein (MYC) and apoptosis regulator BCL‐2 was observed. BCL‐2 was upregulated in basal condition (*p* value < 0.001), while MYC expression was upregulated in paclitaxel‐treated cells (*p* value < 0.001). MYC has a dual action, being involved in both apoptosis and hyperproliferation. Unchecked MYC expression is a hallmark of uncontrolled cell proliferation associated with most forms of cancer [40]. Tumors learned to overexpress MYC (through gene amplification or translocation), evading MYC‐driven apoptosis and activating BCL‐2 [41]. Essentially, cancer cells tolerate high MYC levels by relying on BCL‐2's prosurvival action to blunt apoptotic effects. This suggests that in normal conditions, Caco‐2 cells produce high levels of BCL‐2 to counterbalance MYC overexpression, leading to hyperproliferation. Paclitaxel disrupted this balance by further enhancing MYC expression while simultaneously reducing BCL‐2 levels in treated cells. Paclitaxel is widely known for targeting the β subunit of tubulin (TUBB1), inhibiting microtubule formation during mitosis. However, it also directly targets BCL‐2 due to molecular similarities between paclitaxel‐binding sites on BCL‐2 and β‐tubulin [42]. The induction of the apoptosis pathway by paclitaxel was further confirmed by the upregulation of additional apoptosis biomarkers, including the cellular tumor antigen p53 (a downstream product of the MYC‐driven apoptosis cascade), the tumor necrosis factor‐related apoptosis‐inducing ligand 10 (TRAIL), and the TNFRSF10C. TRAIL plays an important role in programmed cell death and tumor immunosurveillance [23]. It selectively induces apoptosis in tumor cells, making it an attractive target for anticancer compounds aimed at modulating its levels [43]. However, tumor cells frequently develop resistance mechanisms to TRAIL, limiting its clinical potential [44]. To evaluate tumor strategies for overcoming TRAIL upregulation, we monitored TNFRSF10C, an antagonist decoy receptor that lacks a cytoplasmic death domain, hijacking apoptosis induction by competing with TRAIL‐R1 and R2 receptors for ligand binding. Caco‐2 cells treated with paclitaxel showed an upregulation of both TRAIL and the decoy receptor TNFRSF10C (*p* value < 0.01 in both cases). This final evidence indicated a defense strategy adopted by tumor cells to escape cell death, even in the presence of paclitaxel‐induced TRAIL expression.

Overall, the pairwise comparison between a well‐known chemotherapy agent (paclitaxel) and the normal growth conditions of tumor cells (basal medium) provided a clear reference point for the initial evaluation of the cellular effects induced by the R and RO peptides (Table S9). Both R and RO peptides upregulated the expression of FASL and FAS (*p* value < 0.05). However, R peptides induced a higher upregulation of FAS compared to RO peptides (*p* value < 0.01). Unexpectedly, only RO peptides triggered a statistically significant overexpression of CASP2 relative to cells in basal medium (*p* value < 0.05). The absence of caspase activation in response to R peptides may indicate a different cellular response, which has not yet been investigated. The FAS/FASL ligand pathway is not exclusively associated with caspase activation; its role in pathology has been expanded by the finding that FAS receptor upregulation can be induced by cellular lesions and DNA damage [45]. Intriguingly, FAS receptor upregulation appears to be modulated by p53 [45]. Supporting this connection, cells treated with both R and RO peptides showed a significant overexpression of p53 (*p* value < 0.001), suggesting a reasonable correlation between biomarker expression and the phenotype observed during the proliferation assay. In addition to p53 upregulation, RO peptides triggered overexpression of MYC (*p* value < 0.001), indicating another potential apoptosis‐related mechanism. Conversely, BCL‐2 was downregulated in both peptide conditions compared to basal medium (*p* value < 0.001). The antiproliferative effects of RO peptides may therefore involve a combined mechanism of MYC upregulation and BCL‐2 downregulation. R peptides, on the other hand, did not induce significant MYC upregulation (*p* value = 0.09), although the *p* value approached the significance threshold (*p* value ≤ 0.05). Despite this, the observed downregulation of BCL‐2 alone may still contribute to a similar outcome. Despite no defined molecular interaction between BCL‐2 and R/RO peptides can be proposed at this stage, prior research has reported peptide‐mediated impairment of active BCL‐2 homo and heterodimer assemblies, thereby blocking its antiapoptotic action [46]. Such interactions could unbalance gene expression (e.g., feedback inhibition), leading to BCL‐2 downregulation. Additionally, the upregulation of TNFA and TNR1A was examined. Both R and RO peptides upregulated TNFA compared to basal medium (*p* value < 0.05). Similarly, TNR1A was upregulated with a *p* value < 0.05 for R peptides and *p* value < 0.01 for RO peptides. The complex and multifaceted cascade of events induced by the TNF/TNR1 pathway presents a significant challenge in establishing a direct relationship with an univocal cellular response [47]. A tentative explanation is the activation of nuclear transcription factor‐κB (NF‐κB). Although NF‐κB is primarily recognized for its antiapoptotic role, it also interacts with other processes regulating the life‐death balance, such as autophagy, necroptosis, and inflammation [48]. Although we did not collect specific data on NF‐κB activation, the TNF/TNR1 pathway could plausibly contribute to apoptosis and the activation of proinflammatory genes, expressing cytokines and chemokines [49]. The upregulation of TNF/TNFR1 signaling pathway may therefore represent a biological response to cellular damage, providing a potential link between the differential expressions observed by LC‐MS/MS targeted proteomics and the functional assay results.

Finally, corroborating the correlation between data mining and experimental evidence, the groups observed from the heatmap of the apoptosis biomarker study (Figure 5) were consistent with the PLS‐DA analysis of the cytotoxic protein profiles of the different quinoa grain varieties (Figure 3). To further understand the connections between the cellular effects and the quinoa grain proteins, a final chemometric analysis was performed.

#### 3.2.3 Correlation Between Cytotoxic Proteins of Quinoa Grain Varieties, Apoptosis Biomarkers, and Potential Antiproliferative Effect

**FIGURE 6:**
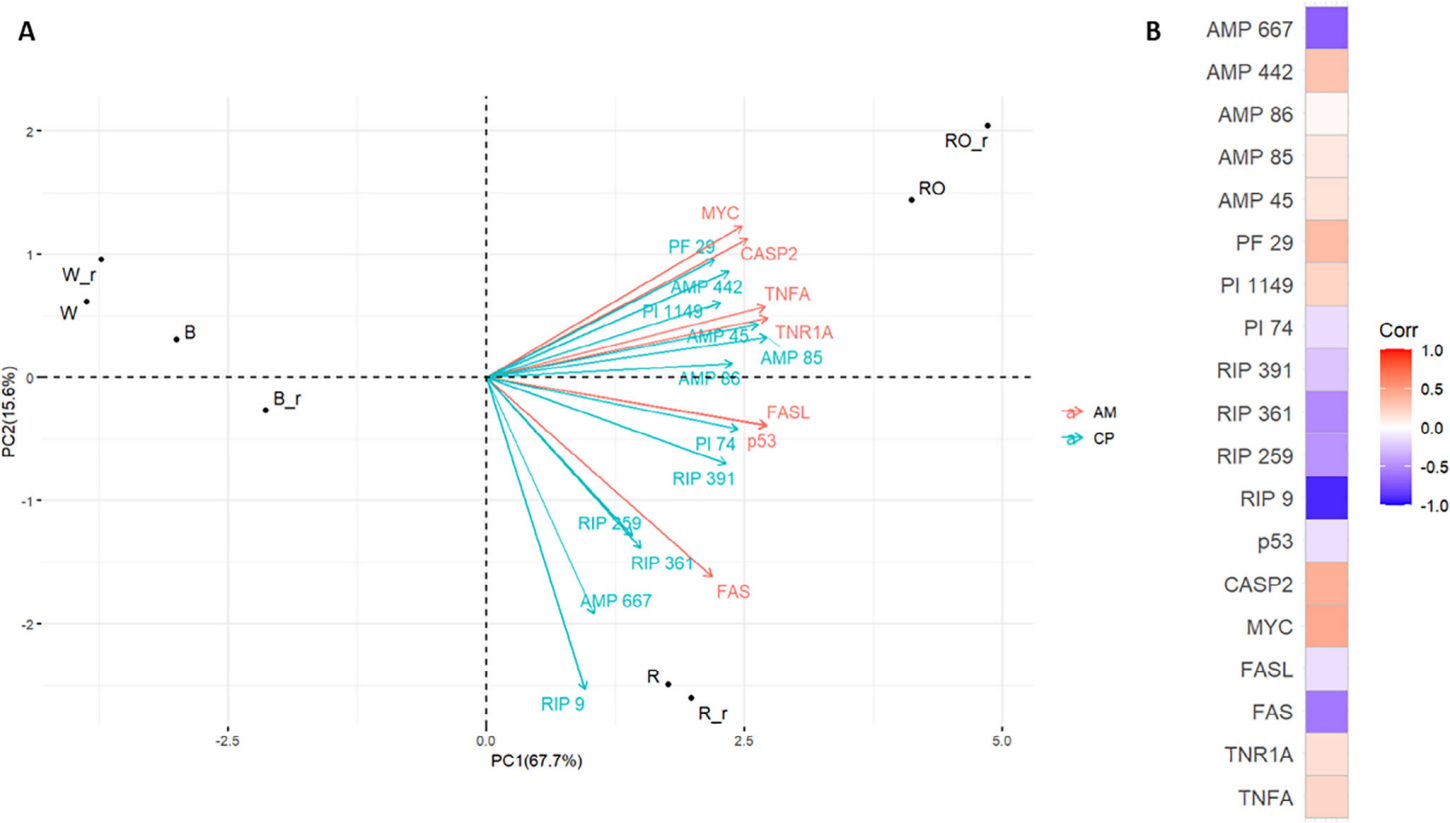
(A) Principal component analysis (PCA) biplot. PCA loadings are highlighted to ease the visualization of AM (apoptosis biomarkers) and CP (cytotoxic proteins). (B) Pearson correlation matrix was calculated to ease the visualization of PCA loadings distribution on PC2. Cytotoxic proteins are listed in Tables S6 and S10 (together with the peak areas of the apoptosis biomarkers). Apoptosis biomarker acronyms are defined as described in Figure 4.

The final chemometric analysis by PCA considered the LFQ intensities of cytotoxic proteins disclosed by data mining and PLS‐DA, with VIP scores greater than 1 and positive LV1 and LV2 (the most relevant for R/RO classification), along with the relative abundances of apoptosis biomarkers that were significantly different compared to the negative control (basal medium) in the R and RO peptide conditions (proapoptotic) (Table S10). Figure 6 shows the PCA biplot and the Pearson correlation matrix.

PCA confirmed our previous results and provided additional insights. As expected, R/RO quinoa grains were both located at positive values of PC1, in contrast to B and W, whose relative abundance of cytotoxic proteins and differential expression of apoptosis biomarkers were remarkably different from the R/RO class. In addition to this confirmation, a clear separation was observed along PC2, suggesting differences at the level of cytotoxic proteins and apoptosis biomarkers. To facilitate data interpretation, a Pearson correlation matrix was used. For positive values of PC2 (RO domain), a positive correlation was observed between the relative abundances of several AMPs, PFT, and PI cytotoxic proteins and the upregulation of apoptosis biomarkers such as TNFA, CASP2, MYC, and TNR1A. Conversely, for negative values of PC2 (R domain), the relative abundances of an AMP and several RIPs were primarily correlated to the upregulation of FAS. This data analysis suggests that differences in cytotoxic protein profiles may result in similar in vitro cellular effects induced by different mechanisms, providing a foundation for further investigation. For instance, isolating and testing the identified cytotoxic proteins or enriched fractions directly on Caco‐2 cells could not only validate their apoptosis‐related effects but also provide a better understanding of the molecular mechanisms involved.

## 4 Concluding Remarks

This study demonstrated the potential of proteins and peptides derived from quinoa grain varieties, particularly R and RO, to induce apoptosis in CRC cells (Caco‐2). Using an integrated approach combining functional assays, LC‐MS/MS proteomics, data mining, and multivariate data analysis, we identified specific cytotoxic proteins, including AMPs, RIPs, PFTs, and PIs, as key contributors to the observed proapoptotic effects. The clustering of R and RO varieties based on their cytotoxic protein profiles, along with a significant upregulation of apoptosis biomarkers, provided strong evidence that proteins and peptides from these quinoa grain varieties held promise as potential anticancer agents. Furthermore, this study underscored the utility of data‐driven and knowledge‐based approaches to generate robust, well‐defined hypotheses about the bioactivity of complex natural mixtures, thereby facilitating the future development of functional foods and nutraceuticals with health‐promoting properties. However, this work was not without limitations. It relied on in vitro models, which, while informative and compatible with advanced 3D models (colonoids), did not fully replicate the complexity of CRC in vivo. In addition, although the exploratory analysis provided valuable insights, the specific mechanism by which these proteins and peptides induced apoptosis needs further investigation and validation through complementary experiments.

## Conflicts of Interest

The authors declare no conflicts of interest.

## Supporting information



Supporting information

Supporting information

## Data Availability

Raw and processed LC‐MS/MS data for the targeted proteomics study are available at Zenodo [50]. The remaining data supporting this article are available in the main manuscript text and as part of the Supporting Information.
