# Supplementary material for: Comparative LC‐MS Proteomics of Quinoa Grains: Evaluation of Bioactivity and Health Benefits by Combining In Silico Techniques With In Vitro Assays on Colorectal Adenocarcinoma Cells
Source: Mol Nutr Food Res. 2025 May 23;69(14):e70125. doi: 10.1002/mnfr.70125 (PMC12280847; doi:10.1002/mnfr.70125)
Supplement: Supplementary file 2 — Supporting information [file MNFR-69-e70125-s001.docx]

**Supplementary Materials**

**Comparative LC-MS proteomics of quinoa grains: evaluation of bioactivity and health benefits by combining *in silico* techniques with *in vitro* assays on colorectal adenocarcinoma cells**

Alessandro Zaccarelli^1^. Beatrice Mattina^1^. Rocío Galindo-Luján^2^. Laura Pont^2,3^. Fernando Benavente^2*^. Ilaria Zanotti^1^. Lisa Elviri^1*^

^1^Department of Food and Drug. University of Parma. 43124 Parma. Italy.

^2^Department of Chemical Engineering and Analytical Chemistry. Institute for Research on Nutrition and Food Safety (INSA·UB). University of Barcelona. 08028. Barcelona. Spain

^3^Serra Húnter Program. Generalitat de Catalunya. 08007. Barcelona. Spain

^*^Corresponding authors: lisa.elviri@unipr.it (L. Elviri. PhD)

Tel: (+39) 0521905030

fbenavente@ub.edu (F. Benavente. PhD)

Tel: (+34) 934021796

**MS/MS transitions and instrumental parameters set for the targeted LC-MS/MS proteomic analysis of apoptosis biomarkers.**

| **Protein biomarker** | **Peptide Modified Sequence** | **Precursor m/z** | **Collision Energy (a.u.)** | **Product m/z** | **Fragment Ion** |
| --- | --- | --- | --- | --- | --- |
| TNFA | DLSLISPLAQAVR | 692.3142 | 32.9 | 841.9798 | y8 |
| TNFA | DLSLISPLAQAVR | 692.3142 | 32.9 | 754.9021 | y7 |
| TNFA | DLSLISPLAQAVR | 692.3142 | 32.9 | 629.7264 | b6 |
| TNFA | DLSLISPLAQAVR | 692.3142 | 32.9 | 726.8423 | b7 |
| TNFA | DLSLISPLAQAVR | 692.3142 | 32.9 | 840.0009 | b8 |
| TNFA | ANALLANGVELR | 621.2159 | 29.4 | 758.8474 | y7 |
| TNFA | ANALLANGVELR | 621.2159 | 29.4 | 687.7691 | y6 |
| TNFA | ANALLANGVELR | 621.2159 | 29.4 | 668.7659 | b7 |
| TNFA | ANALLANGVELR | 621.2159 | 29.4 | 725.8175 | b8 |
| TNFA | VNLLSAIK | 429.5349 | 20 | 644.8275 | y6 |
| TNFA | VNLLSAIK | 429.5349 | 20 | 531.6689 | y5 |
| TNFA | VNLLSAIK | 429.5349 | 20 | 440.5594 | b4 |
| TNFA | VNLLSAIK | 429.5349 | 20 | 527.6372 | b5 |
| TNFRSF1A | EMGQVEISSC[+57.1]TVDR | 806.3947 | 38.5 | 938.0424 | y8 |
| TNFRSF1A | EMGQVEISSC[+57.1]TVDR | 806.3947 | 38.5 | 824.8839 | y7 |
| TNFRSF1A | EMGQVEISSC[+57.1]TVDR | 806.3947 | 38.5 | 874.9832 | b8 |
| TNFRSF1A | EMGQVEISSC[+57.1]TVDR | 806.3947 | 38.5 | 962.0610 | b9 |
| TNFRSF1A | LC[+57.1]LPQIENVK | 607.7346 | 28.8 | 827.9500 | y7 |
| TNFRSF1A | LC[+57.1]LPQIENVK | 607.7346 | 28.8 | 730.8340 | y6 |
| TNFRSF1A | LC[+57.1]LPQIENVK | 607.7346 | 28.8 | 387.5193 | b3 |
| TNFRSF1A | LC[+57.1]LPQIENVK | 607.7346 | 28.8 | 612.7652 | b5 |
| TNFRSF1A | LC[+57.1]LPQIENVK | 607.7346 | 28.8 | 725.9238 | b6 |
| TNFRSF1A | EAQYSMLATWR | 678.7708 | 32.3 | 865.0366 | y7 |
| TNFRSF1A | EAQYSMLATWR | 678.7708 | 32.3 | 777.9589 | y6 |
| TNFRSF1A | EAQYSMLATWR | 678.7708 | 32.3 | 710.7795 | b6 |
| TNFRSF1A | EAQYSMLATWR | 678.7708 | 32.3 | 823.9380 | b7 |
| FAS | EC[+57.1]TLTSNTK | 527.5828 | 24.9 | 663.7442 | y6 |
| FAS | EC[+57.1]TLTSNTK | 527.5828 | 24.9 | 550.5857 | y5 |
| FAS | EC[+57.1]TLTSNTK | 527.5828 | 24.9 | 605.6845 | b5 |
| FAS | EC[+57.1]TLTSNTK | 527.5828 | 24.9 | 692.7622 | b6 |
| FAS | NDNVQDTAEQK | 631.6374 | 30 | 819.8412 | y7 |
| FAS | NDNVQDTAEQK | 631.6374 | 30 | 691.7113 | y6 |
| FAS | NDNVQDTAEQK | 631.6374 | 30 | 686.6515 | b6 |
| FAS | NDNVQDTAEQK | 631.6374 | 30 | 787.7560 | b7 |
| FAS | DITSDSENSNFR | 693.1926 | 33 | 853.8611 | y7 |
| FAS | DITSDSENSNFR | 693.1926 | 33 | 766.7833 | y6 |
| FAS | DITSDSENSNFR | 693.1926 | 33 | 748.7165 | b7 |
| FAS | DITSDSENSNFR | 693.1926 | 33 | 862.8198 | b8 |
| FASL | QIGHPSPPPEK | 594.1698 | 28.1 | 751.8519 | y7 |
| FASL | QIGHPSPPPEK | 594.1698 | 28.1 | 654.7360 | y6 |
| FASL | QIGHPSPPPEK | 594.1698 | 28.1 | 567.6583 | y5 |
| FASL | QIGHPSPPPEK | 594.1698 | 28.1 | 470.5423 | y4 |
| FASL | QIGHPSPPPEK | 594.1698 | 28.1 | 373.4264 | y3 |
| FASL | QIGHPSPPPEK | 594.1698 | 28.1 | 436.4876 | b4 |
| FASL | QIGHPSPPPEK | 594.1698 | 28.1 | 620.6813 | b6 |
| FASL | QIGHPSPPPEK | 594.1698 | 28.1 | 717.7972 | b7 |
| FASL | QIGHPSPPPEK | 594.1698 | 28.1 | 814.9131 | b8 |
| FASL | GGLVINETGLYFVYSK | 881.0109 | 42.2 | 977.1399 | y8 |
| FASL | GGLVINETGLYFVYSK | 881.0109 | 42.2 | 920.0883 | y7 |
| FASL | GGLVINETGLYFVYSK | 881.0109 | 42.2 | 955.0920 | b10 |
| FASL | GGLVINETGLYFVYSK | 881.0109 | 42.2 | 1118.2666 | b11 |
| FASL | YPQDLVMMEGK | 656.2809 | 31.2 | 1148.3800 | y10 |
| FASL | YPQDLVMMEGK | 656.2809 | 31.2 | 808.0461 | y7 |
| FASL | YPQDLVMMEGK | 656.2809 | 31.2 | 694.8875 | y6 |
| FASL | YPQDLVMMEGK | 656.2809 | 31.2 | 716.8061 | b6 |
| FASL | YPQDLVMMEGK | 656.2809 | 31.2 | 848.0029 | b7 |
| MYC | C[+57.1]HVSTHQHNYAAPPSTR | 982.5623 | 47.1 | 1242.3279 | y11 |
| MYC | C[+57.1]HVSTHQHNYAAPPSTR | 982.5623 | 47.1 | 1114.1980 | y10 |
| MYC | C[+57.1]HVSTHQHNYAAPPSTR | 982.5623 | 47.1 | 557.6232 | y5 |
| MYC | C[+57.1]HVSTHQHNYAAPPSTR | 982.5623 | 47.1 | 460.5073 | y4 |
| MYC | C[+57.1]HVSTHQHNYAAPPSTR | 982.5623 | 47.1 | 988.0668 | b8 |
| MYC | C[+57.1]HVSTHQHNYAAPPSTR | 982.5623 | 47.1 | 1102.1700 | b9 |
| MYC | C[+57.1]HVSTHQHNYAAPPSTR | 982.5623 | 47.1 | 1407.5013 | b12 |
| MYC | DQIPELENNEK | 665.2023 | 31.6 | 973.0209 | y8 |
| MYC | DQIPELENNEK | 665.2023 | 31.6 | 875.9049 | y7 |
| MYC | DQIPELENNEK | 665.2023 | 31.6 | 746.7902 | y6 |
| MYC | DQIPELENNEK | 665.2023 | 31.6 | 357.3838 | b3 |
| MYC | DQIPELENNEK | 665.2023 | 31.6 | 696.7730 | b6 |
| MYC | DQIPELENNEK | 665.2023 | 31.6 | 825.8877 | b7 |
| TNFRSF10C | QEEVPQQTVAPQQQR | 883.9520 | 42.3 | 1281.4055 | y11 |
| TNFRSF10C | QEEVPQQTVAPQQQR | 883.9520 | 42.3 | 1056.1596 | y9 |
| TNFRSF10C | QEEVPQQTVAPQQQR | 883.9520 | 42.3 | 928.0296 | y8 |
| TNFRSF10C | QEEVPQQTVAPQQQR | 883.9520 | 42.3 | 656.7150 | y5 |
| TNFRSF10C | QEEVPQQTVAPQQQR | 883.9520 | 42.3 | 486.4985 | b4 |
| TNFRSF10C | QEEVPQQTVAPQQQR | 883.9520 | 42.3 | 940.9789 | b8 |
| TNFRSF10C | QEEVPQQTVAPQQQR | 883.9520 | 42.3 | 1040.1107 | b9 |
| TNFRSF10C | QEEVPQQTVAPQQQR | 883.9520 | 42.3 | 1111.1890 | b10 |
| TNFRSF10C | GEEC[+57.1]PAGSHR | 550.5781 | 26 | 784.8679 | y7 |
| TNFRSF10C | GEEC[+57.1]PAGSHR | 550.5781 | 26 | 624.6730 | y6 |
| TNFRSF10C | GEEC[+57.1]PAGSHR | 550.5781 | 26 | 476.4833 | b4 |
| TNFRSF10C | GEEC[+57.1]PAGSHR | 550.5781 | 26 | 573.5993 | b5 |
| TNFRSF10C | GEEC[+57.1]PAGSHR | 550.5781 | 26 | 644.6776 | b6 |
| TNFRSF10C | NENSPEMC[+57.1]R | 569.1189 | 26.9 | 692.8316 | y5 |
| TNFRSF10C | NENSPEMC[+57.1]R | 569.1189 | 26.9 | 595.7156 | y4 |
| TNFRSF10C | NENSPEMC[+57.1]R | 569.1189 | 26.9 | 445.4062 | b4 |
| TNFRSF10C | NENSPEMC[+57.1]R | 569.1189 | 26.9 | 671.6369 | b6 |
| TNFRSF10C | NENSPEMC[+57.1]R | 569.1189 | 26.9 | 802.8337 | b7 |
| APAF1 | LPLNIEEAK | 514.1028 | 24.2 | 914.0398 | y8 |
| APAF1 | LPLNIEEAK | 514.1028 | 24.2 | 703.7653 | y6 |
| APAF1 | LPLNIEEAK | 514.1028 | 24.2 | 589.6621 | y5 |
| APAF1 | LPLNIEEAK | 514.1028 | 24.2 | 551.7021 | b5 |
| APAF1 | LPLNIEEAK | 514.1028 | 24.2 | 680.8168 | b6 |
| APAF1 | TLISSSDDAEIQVWNWQLDK | 1175.2750 | 56.6 | 1330.5189 | y10 |
| APAF1 | TLISSSDDAEIQVWNWQLDK | 1175.2750 | 56.6 | 1217.3604 | y9 |
| APAF1 | TLISSSDDAEIQVWNWQLDK | 1175.2750 | 56.6 | 1261.3197 | b12 |
| APAF1 | TLISSSDDAEIQVWNWQLDK | 1175.2750 | 56.6 | 1360.4515 | b13 |
| BCL-2 | DFAEMSSQLHLTPFTAR | 976.5953 | 46.9 | 1184.3748 | y10 |
| BCL-2 | DFAEMSSQLHLTPFTAR | 976.5953 | 46.9 | 1056.2448 | y9 |
| BCL-2 | DFAEMSSQLHLTPFTAR | 976.5953 | 46.9 | 591.6831 | y5 |
| BCL-2 | DFAEMSSQLHLTPFTAR | 976.5953 | 46.9 | 1010.1044 | b9 |
| BCL-2 | DFAEMSSQLHLTPFTAR | 976.5953 | 46.9 | 1147.2446 | b10 |
| BCL-2 | DFAEMSSQLHLTPFTAR | 976.5953 | 46.9 | 1361.5076 | b12 |
| BCL-2 | FATVVEELFR | 606.2006 | 28.7 | 792.9042 | y6 |
| BCL-2 | FATVVEELFR | 606.2006 | 28.7 | 693.7723 | y5 |
| BCL-2 | FATVVEELFR | 606.2006 | 28.7 | 647.7437 | b6 |
| BCL-2 | FATVVEELFR | 606.2006 | 28.7 | 776.8584 | b7 |
| CASP2 | GLALVLSNVHFTGEK | 793.4197 | 37.9 | 932.0170 | y8 |
| CASP2 | GLALVLSNVHFTGEK | 793.4197 | 37.9 | 817.9138 | y7 |
| CASP2 | GLALVLSNVHFTGEK | 793.4197 | 37.9 | 868.0575 | b9 |
| CASP2 | GLALVLSNVHFTGEK | 793.4197 | 37.9 | 1005.1977 | b10 |
| CASP2 | GSWYIEALAQVFSER | 878.9750 | 42.1 | 1021.1538 | y9 |
| CASP2 | GSWYIEALAQVFSER | 878.9750 | 42.1 | 950.0755 | y8 |
| CASP2 | GSWYIEALAQVFSER | 878.9750 | 42.1 | 921.0330 | b8 |
| CASP2 | GSWYIEALAQVFSER | 878.9750 | 42.1 | 992.1113 | b9 |
| CASP2 | EGYAPGTEFHR | 632.6689 | 30 | 843.9113 | y7 |
| CASP2 | EGYAPGTEFHR | 632.6689 | 30 | 746.7954 | y6 |
| CASP2 | EGYAPGTEFHR | 632.6689 | 30 | 689.7437 | y5 |
| CASP2 | EGYAPGTEFHR | 632.6689 | 30 | 421.4266 | b4 |
| CASP2 | EGYAPGTEFHR | 632.6689 | 30 | 676.6986 | b7 |
| CASP2 | EGYAPGTEFHR | 632.6689 | 30 | 805.8133 | b8 |
| CASP3 | EEIVELMR | 510.1032 | 24 | 647.8110 | y5 |
| CASP3 | EEIVELMR | 510.1032 | 24 | 548.6792 | y4 |
| CASP3 | EEIVELMR | 510.1032 | 24 | 600.6418 | b5 |
| CASP3 | EEIVELMR | 510.1032 | 24 | 713.8004 | b6 |
| CASP3 | LEFMHILTR | 580.7117 | 27.5 | 770.9677 | y6 |
| CASP3 | LEFMHILTR | 580.7117 | 27.5 | 639.7709 | y5 |
| CASP3 | LEFMHILTR | 580.7117 | 27.5 | 658.7927 | b5 |
| CASP3 | LEFMHILTR | 580.7117 | 27.5 | 771.9513 | b6 |
| CASP3 | VATEFESFSFDATFHAK | 968.0464 | 46.4 | 1171.2885 | y10 |
| CASP3 | VATEFESFSFDATFHAK | 968.0464 | 46.4 | 1024.1133 | y9 |
| CASP3 | VATEFESFSFDATFHAK | 968.0464 | 46.4 | 999.0573 | b9 |
| CASP3 | VATEFESFSFDATFHAK | 968.0464 | 46.4 | 1146.2325 | b10 |
| CASP8 | C[+57.1]PSLAGKPK | 479.5846 | 22.5 | 797.9670 | y8 |
| CASP8 | C[+57.1]PSLAGKPK | 479.5846 | 22.5 | 613.7734 | y6 |
| CASP8 | C[+57.1]PSLAGKPK | 479.5846 | 22.5 | 500.6148 | y5 |
| CASP8 | C[+57.1]PSLAGKPK | 479.5846 | 22.5 | 529.6328 | b5 |
| CASP8 | C[+57.1]PSLAGKPK | 479.5846 | 22.5 | 586.6844 | b6 |
| CASP8 | C[+57.1]PSLAGKPK | 479.5846 | 22.5 | 714.8576 | b7 |
| CASP8 | GDDILTILTEVNYEVSNK | 1012.6128 | 48.6 | 1183.2521 | y10 |
| CASP8 | GDDILTILTEVNYEVSNK | 1012.6128 | 48.6 | 1082.1477 | y9 |
| CASP8 | GDDILTILTEVNYEVSNK | 1012.6128 | 48.6 | 1072.1927 | b10 |
| CASP8 | GDDILTILTEVNYEVSNK | 1012.6128 | 48.6 | 1171.3246 | b11 |
| p53 | TYQGSYGFR | 540.0781 | 25.5 | 629.6883 | y5 |
| p53 | TYQGSYGFR | 540.0781 | 25.5 | 542.6106 | y4 |
| p53 | TYQGSYGFR | 540.0781 | 25.5 | 700.7203 | b6 |
| p53 | TYQGSYGFR | 540.0781 | 25.5 | 757.7719 | b7 |
| p53 | SVTC[+57.1]TYSPALNK | 671.2625 | 31.9 | 894.0086 | y8 |
| p53 | SVTC[+57.1]TYSPALNK | 671.2625 | 31.9 | 792.9042 | y7 |
| p53 | SVTC[+57.1]TYSPALNK | 671.2625 | 31.9 | 542.6518 | y5 |
| p53 | SVTC[+57.1]TYSPALNK | 671.2625 | 31.9 | 712.7954 | b6 |
| p53 | SVTC[+57.1]TYSPALNK | 671.2625 | 31.9 | 799.8731 | b7 |
| p53 | QSQHMTEVVR | 608.1870 | 28.8 | 872.0290 | y7 |
| p53 | QSQHMTEVVR | 608.1870 | 28.8 | 734.8888 | y6 |
| p53 | QSQHMTEVVR | 608.1870 | 28.8 | 612.6820 | b5 |
| p53 | QSQHMTEVVR | 608.1870 | 28.8 | 713.7864 | b6 |
| p53 | ELNEALELK | 530.1022 | 25 | 702.8206 | y6 |
| p53 | ELNEALELK | 530.1022 | 25 | 573.7059 | y5 |
| p53 | ELNEALELK | 530.1022 | 25 | 557.5769 | b5 |
| p53 | ELNEALELK | 530.1022 | 25 | 670.7354 | b6 |
| TRAIL | TSEETISTVQEK | 676.7182 | 32.2 | 804.9130 | y7 |
| TRAIL | TSEETISTVQEK | 676.7182 | 32.2 | 691.7545 | y6 |
| TRAIL | TSEETISTVQEK | 676.7182 | 32.2 | 748.7597 | b7 |
| TRAIL | TSEETISTVQEK | 676.7182 | 32.2 | 849.8642 | b8 |
| TRAIL | NGELVIHEK | 520.0882 | 24.5 | 625.7410 | y5 |
| TRAIL | NGELVIHEK | 520.0882 | 24.5 | 526.6092 | y4 |
| TRAIL | NGELVIHEK | 520.0882 | 24.5 | 626.7258 | b6 |
| TRAIL | NGELVIHEK | 520.0882 | 24.5 | 763.8659 | b7 |
| TRAIL | DAEYGLYSIYQGGIFELK | 1034.1473 | 49.7 | 1168.3691 | y10 |
| TRAIL | DAEYGLYSIYQGGIFELK | 1034.1473 | 49.7 | 1055.2106 | y9 |
| TRAIL | DAEYGLYSIYQGGIFELK | 1034.1473 | 49.7 | 1176.2586 | b10 |
| TRAIL | DAEYGLYSIYQGGIFELK | 1034.1473 | 49.7 | 1304.3886 | b11 |

**Supplementary Table S7.** MS/MS transitions and instrumental parameters set for the targeted LC-MS/MS proteomic analysis of Caco-2 apoptosis biomarkers. Skyline was used to obtain the MS/MS transition of peptides and to optimize collision energy. Peptide fragment ions are indicated according to Bienmann nomenclature. Apoptosis biomarkers are listed as acronyms: Tumor Necrosis Factor α (TNFA), Tumor Necrosis Factor Receptor 1A (TNR1A), Tumor Necrosis Factor Receptor 6 (FAS), its ligand (FASL), CASPASE 2,3,8 (CASP 2-3-8), Apoptotic Protease Activating Factor-1 (APAF1), proto-oncogene protein (MYC), B-Cell Lymphoma 2 (BCL-2), cellular tumor antigen (p53), Tumor Necrosis Factor-Related Apoptosis-Inducing Ligand 10 (TRAIL), Tumor Necrosis Factor Receptor 10C (TNFRSF10C).

**LC-MS/MS peak areas of the apoptosis biomarkers.**

| **Apoptosis biomarker** | **basal** | **taxol** | **RO pep** | **R pep** | **W pep** | **B pep** | **RO pro** | **R pro** | **RSD %** |
| --- | --- | --- | --- | --- | --- | --- | --- | --- | --- |
| TNFA | 0.39 | 11.60 | 3.69 | 2.18 | 0.85 | 0.93 | 1.10 | 0.84 | 11.40 |
| TNR1A | 0.18 | 4.10 | 2.78 | 1.65 | 0.50 | 0.56 | 0.65 | 0.52 | 10.10 |
| FAS | 0.21 | 8.12 | 3.02 | 4.66 | 0.87 | 0.94 | 0.87 | 1.02 | 10.30 |
| FASL | 0.46 | 7.47 | 6.37 | 5.50 | 0.69 | 0.70 | 1.05 | 0.86 | 9.90 |
| MYC | 0.27 | 6.31 | 3.63 | 1.39 | 0.71 | 0.80 | 0.85 | 0.51 | 12.50 |
| TNFRSF10C | 0.36 | 4.50 | 0.94 | 1.02 | 0.72 | 0.75 | 0.54 | 0.48 | 13.10 |
| APAF1 | 0.21 | 6.50 | 0.22 | 0.25 | 0.29 | 0.30 | 0.31 | 0.32 | 11.90 |
| BCL-2 | 3.55 | 0.35 | 0.33 | 0.33 | 0.30 | 0.30 | 0.27 | 0.27 | 9.70 |
| CASP2 | 0.19 | 6.55 | 0.5 | 0.25 | 0.18 | 0.19 | 0.17 | 0.16 | 10.30 |
| CASP3 | 0.19 | 5.65 | 0.23 | 0.32 | 0.25 | 0.27 | 0.22 | 0.31 | 12.70 |
| CASP8 | 0.20 | 8.15 | 0.26 | 0.22 | 0.20 | 0.21 | 0.25 | 0.28 | 12.90 |
| p53 | 1.34 | 10.00 | 10.94 | 9.63 | 2.11 | 2.14 | 2.58 | 2.54 | 9.80 |
| TRAIL | 0.08 | 3.00 | 0.08 | 0.09 | 0.07 | 0.07 | 0.07 | 0.07 | 13.50 |

**Supplementary Table S8.** LC-MS/MS peak areas of the apoptosis biomarkers and average (n = 2) percent Relative Standard Deviation (RSD %). Peak areas were normalized against the total protein content measured by the BCA assay.

**MSstats pairwise group comparison between controls and R/RO quinoa peptides.**

| **Apoptosis biomarkers** | **basal vs taxol** | **basal vs RO pep** | **basal vs R pep** | **taxol vs RO pep** | **taxol vs R pep** |
| --- | --- | --- | --- | --- | --- |
| TNFA | 0.008 | 0.02 | 0.04 | < 0.001 | < 0.001 |
| TNR1A | 0.002 | 0.002 | 0.02 | < 0.001 | 0.01 |
| FAS | 0.005 | 0.02 | < 0.01 | < 0.001 | < 0.001 |
| FASL | 0.002 | < 0.01 | < 0.01 | 0.03 | 0.002 |
| MYC | < 0.001 | < 0.001 | 0.09 | < 0.001 | 0.03 |
| TNFRSF10C | < 0.01 | 0.06 | 0.08 | < 0.001 | < 0.01 |
| APAF1 | < 0.001 | 0.4 | 0.5 | < 0.001 | < 0.05 |
| BCL-2 | < 0.001 | < 0.001 | < 0.001 | 0.25 | 0.3 |
| CASP2 | < 0.001 | 0.02 | 0.2 | < 0.001 | < 0.01 |
| CASP3 | 0.001 | 0.4 | 0.25 | < 0.001 | < 0.01 |
| CASP8 | < 0.001 | 0.14 | 0.9 | < 0.001 | < 0.01 |
| p53 | < 0.001 | 0.0005 | 0.0009 | 0.01 | 0.02 |
| TRAIL | < 0.01 | 0.35 | 0.5 | 0.01 | 0.01 |

**Supplementary Table S9.** Adjusted *p*-values (Benjamini-Hochberg procedure) from pairwise group comparison results of positive (basal) and negative (taxol) controls. along with the statistical analysis of R and RO peptides against controls. The only apoptosis biomarker (BCL-2) up-regulated in basal medium for all reported conditions is highlighted in light blue. Cells highlighted in yellow reported up-regulated apoptosis biomarkers for quinoa peptide conditions compared to positive and negative controls of proliferation.

**LFQ intensities of the most important (VIP > 1) cytotoxic proteins for the classification task combined with LC-MS/MS peak areas of apoptosis biomarkers significantly expressed between the most active peptides (R/RO) and positive control of proliferation.**

| **Proteins** | **W** | **W_r** | **B** | **B_r** | **R** | **R_r** | **RO** | **RO_r** | **type** |
| --- | --- | --- | --- | --- | --- | --- | --- | --- | --- |
| TNFA | 0.87 | 0.83 | 0.93 | 0.92 | 2.16 | 2.2 | 3.72 | 3.65 | AM |
| TNR1A | 0.48 | 0.51 | 0.55 | 0.57 | 1.7 | 1.59 | 2.78 | 2.77 | AM |
| FAS | 0.81 | 0.92 | 0.89 | 0.98 | 4.65 | 4.66 | 3.01 | 3.02 | AM |
| FASL | 0.73 | 0.65 | 0.71 | 0.69 | 5.4 | 5.6 | 6.32 | 6.41 | AM |
| MYC | 0.73 | 0.69 | 0.81 | 0.79 | 1.39 | 1.38 | 3.64 | 3.62 | AM |
| CASP2 | 0.18 | 0.18 | 0.17 | 0.19 | 0.26 | 0.27 | 0.5 | 0.51 | AM |
| p53 | 2.1 | 2.12 | 2.15 | 2.13 | 9.75 | 9.5 | 10.8 | 11.08 | AM |
| XP_021717218.1 heat shock 70 kDa protein, mitochondrial-like | 0.02 | 0.02 | 0.02 | 0.02 | 0.02 | 0.02 | 0.02 | 0.02 | CP |
| XP_021767383.1 UDP-D-apiose/UDP-D-xylose synthase 2 | 0.01 | 0.01 | 0.01 | 0.01 | 0.01 | 0.01 | 0.01 | 0.01 | CP |
| XP_021756776.1 heat shock 70 kDa protein-like | 0.02 | 0.02 | 0.03 | 0.02 | 0.03 | 0.03 | 0.03 | 0.02 | CP |
| XP_021774970.1 luminal-binding protein | 0.00 | 0.01 | 0.01 | 0.01 | 0.02 | 0.02 | 0.02 | 0.02 | CP |
| XP_021762341.1 cysteine proteinase inhibitor 6-like | 198230000.00 | 210430000.00 | 196700000.00 | 263280000.00 | 258240000.00 | 281980000.00 | 269260000.00 | 296250000.00 | CP |
| XP_021768406.1 lipid transfer-like protein VAS | 10641000.00 | 11701000.00 | 14074000.00 | 15514000.00 | 18331000.00 | 18928000.00 | 18156000.00 | 34476000.00 | CP |
| XP_021732234.1 uncharacterized protein LOC110699051 | 104710000.00 | 119160000.00 | 84677000.00 | 81774000.00 | 126630000.00 | 112050000.00 | 163790000.00 | 151630000.00 | CP |
| XP_021742662.1 protein disulfide-isomerase like 2-1-like; XP_021772768.1 protein disulfide-isomerase like 2-1-like | 44907000.00 | 52401000.00 | 61597000.00 | 62032000.00 | 67285000.00 | 72768000.00 | 82232000.00 | 91412000.00 | CP |
| XP_021768828.1 legumin A-like | 4175400000.00 | 3855100000.00 | 4632300000.00 | 4675100000.00 | 5511000000.00 | 5410800000.00 | 6535000000.00 | 6568100000.00 | CP |
| XP_021768838.1 11S globulin seed storage protein 2-like | 6245000000.00 | 5735700000.00 | 7469500000.00 | 7260800000.00 | 7491500000.00 | 7540700000.00 | 8101900000.00 | 8514400000.00 | CP |
| XP_021747589.1 vicilin-like antimicrobial peptides 2-3 | 127790000.00 | 134190000.00 | 86111000.00 | 100730000.00 | 139980000.00 | 152520000.00 | 185460000.00 | 206240000.00 | CP |
| XP_021735259.1 protein disulfide isomerase-like 2-3; XP_021724558.1 protein disulfide isomerase-like 2-3 | 30020000.00 | 23610000.00 | 23888000.00 | 23780000.00 | 33883000.00 | 32910000.00 | 25837000.00 | 27567000.00 | CP |

**Supplementary Table S10.** LFQ intensities from all quinoa grains of cytotoxic proteins with VIP scores greater than 1 and positive LV1 and LV2 (the most relevant for R/RO classification), combined with the LC-MS/MS peak areas of apoptosis biomarkers that were at least significantly different from the positive control (basal medium) in the R and RO peptide conditions (most related to the apoptosis pathway). Data are reported in duplicates (n = 2, where r stands for “replicate”). Acronyms appearing in "type" column indicate AM: apoptosis biomarkers; CP: cytotoxic proteins.
